# Discovering Conserved Properties of Brain Organization Through Multimodal Integration and Interspecies Comparison

**DOI:** 10.1177/1179069519862047

**Published:** 2019-07-09

**Authors:** Ben D Fulcher

**Affiliations:** School of Physics, The University of Sydney, Sydney, NSW 2006, Australia

**Keywords:** neuroinformatics, interspecies comparison, spatial embedding, data integration

## Abstract

The primate cerebral cortex is broadly organized along hierarchical processing streams underpinned by corresponding variation in the brain’s microstructure and interareal connectivity patterns. Fulcher et al. recently demonstrated that a similar organization exists in the mouse cortex by combining independent datasets of cytoarchitecture, gene expression, cell densities, and long-range axonal connectivity. Using the T1w:T2w magnetic resonance imaging map as a common spatial reference for data-driven comparison of cortical gradients between mouse and human, we highlighted a common hierarchical expression pattern of numerous brain-related genes, providing new understanding of how systematic structural variation shapes functional specialization in mammalian brains. Reflecting on these findings, here we discuss how open neuroscience datasets, combined with advanced neuroinformatics approaches, will be crucial in the ongoing search for organization principles of brain structure. We explore the promises and challenges of integrative studies and argue that a tighter collaboration between experimental, statistical, and theoretical neuroscientists is needed to drive progress further.

**Comment on:** Fulcher BD, Murray JD, Zerbi V, Wang XJ. Multimodal gradients across mouse cortex. *Proc Natl Acad Sci U S A*. 2019;116:4689-4695. doi: 10.1073/pnas.1814144116. PubMed PMID:30782826; PubMed Central PMCID:PMC6410879. https://www.ncbi.nlm.nih.gov/pubmed/30782826

A core question in neuroscience is how the brain’s structure enables the rich cognitive function that defines our conscious experience. Clinically, such knowledge would provide a mechanistic basis from which to design treatments to minimize cognitive deficits in disease, or to enhance cognitive function in healthy individuals. Technologically, it may open the door to neuromimicry: brain-inspired design of new algorithmic frameworks for artificial intelligence. Recent unprecedented investments in neuroscience research have yielded large volumes of data ranging from intricate whole-brain maps of the microscopic properties of neural circuits to neural dynamics measured across a wide range of spatial and temporal scales. Despite this deluge of data, we have relatively few organizing principles to explain the patterns, make predictions, and design new experiments to test them.^1–3^ Progress in this direction requires closer collaboration between scientists with experimental, statistical, and theoretical modeling expertise. In this commentary, we highlight key results from our recent paper,^4^ framing them in the context of the modern neuroscience landscape.

## The Prominence of Neuroinformatics on the Landscape of Modern Neuroscience

**Figure 1.**
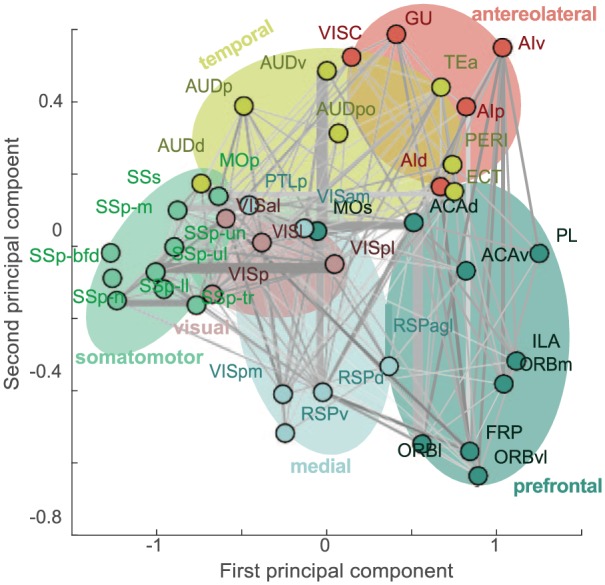
A low-dimensional projection of mouse brain cortical areas, represented as a multimodal feature vector. The feature vector combines properties of gene expression (*Pvalb, Grin3a*, and *Grik2*), cytoarchitecture type, weighted axonal in-degree, T1w:T2w, estimated hierarchical level, and mean density of parvalbumin-containing cells. Brain areas with a similar set of properties are close in this projection space. Shading has been added manually to highlight the different functional families, labeled according to Harris et al.^12^

In Fulcher et al.,^4^ we assembled multiple datasets as spatial maps of the mouse cortex: (1) gene expression,^5^ (2) cell type densities,^6,7^ (3) axonal connectivity,^8^ (4) cytoarchitecture,^9^ and (5) magnetic resonance imaging (MRI) structural maps.^10^ We demonstrated a surprising commonality of spatial variation between these diverse measurements, which together ordered mouse brain areas along a putative functional hierarchy, from primary somatosensory through to transmodal prefrontal areas. Our integrative approach provides clues about the multifaceted structural underpinning of hierarchical functional specialization^11^ and allowed us to produce data-driven cortical organization schemes by representing each cortical area as a multimodal set of properties, such as that shown in Figure 1.

Our study is an example of integrative research that bridges the specialized expertise of many individual laboratories through data sharing. Following pioneering large-scale experimental work by the Allen Institute^5^ and its associated informatics challenges,^13^ the culture of data sharing in neuroscience has become widespread. For computational and theoretical scientists, this provides new opportunities to leverage what are large financial investments in cutting-edge experimental protocols to discover new properties of brain organization, including those that extend across datasets that would not traditionally be compared. This line of research is not without substantial challenges: compared with prototypical collaborations of experimental and theoretical scientists, analyses of open data involve an unusual level of disconnect between the process of data collection and statistical analysis. This requires much greater care on the part of the data analyst to proactively ensure that their analyses respect the subtleties of the dataset and any limitations in its measurement. For example, recent work comparing studies analyzing transcriptional data from the Allen Human Brain Atlas^14^ found a high degree of inconsistency in how researchers subsequently processed the data and demonstrated that these choices can have a large impact on the final results and their interpretation.^15^

## Data-Driven Interspecies Comparison

Properties of brain organization that are shared across scales and species are strong candidates for providing an evolutionary advantage by enabling cost-efficient information processing. Although some such conserved properties are derived from abstractions of the brain as topological networks,^16^ studies of comparative anatomy (most commonly performed between different primate species) require a cortical parcellation mapped between species. Both the choice of parcellation and its mapping require subjective choices on the part of the researcher.^17,18^ In Fulcher et al.,^4^ we circumvented the need to define a mouse-human homology of cortical areas, instead using the noninvasive MRI contrast map, T1w:T2w, as a common spatial reference for interspecies comparison. As well as confirming the increase of T1w:T2w from dysgranular to eulaminate areas in mouse, as in primate and human,^11^ our results revealed a compelling consistency in the transcriptional patterning of brain-related genes relative to T1w:T2w. Mapping ortholog genes between mouse and human, we found significant mouse-human correspondence: Spearman ρ = 0.44 (*P* = 1 × 10^−4^), a result that was reproduced across a wide variety of gene sets: all genes, brain-expressed genes, astrocyte-enriched genes, neuron-enriched genes, and oligodendrocyte-enriched genes. As T1w:T2w is commonly interpreted as an MRI imaging marker for myelin content,^19^ interspecies correspondence may be expected on the basis of common variation of genes coding for myelin content. Whereas myelin-related gene expression did show interspecies correspondence, e.g., *Mobp* (ρ_mouse_ = 0.43, ρ_human_ = 0.41) and *Mbp* (ρ_mouse_ = 0.34, ρ_human_ = 0.45), the overall correlation was driven by functionally diverse genes, including the striking interspecies correspondences of N-methyl-D-aspartate (NMDA) receptor signaling genes such as *Grin3a* (ρ_mouse_ = –0.63, ρ_human_ = –0.65) and the interneuron marker *Pvalb* (ρ_mouse_ = 0.57, ρ_human_ = 0.70).

The agreement is striking given (1) measurement noise in both datasets, (2) differences in measurement modalities between mouse (high-throughput in situ hybridization) and human (post-mortem microarray from 6 adults), and (3) vast differences in spatial scale. Although we developed new quality-control methods for the data, we note that the atlas-based expression measurements can be very noisy and that a noisy measurement in one species would manifest as a reduced correlation with T1w:T2w. In this respect, our data are again consistent with a meaningful interspecies correspondence: a high correlation in one species consistently matched the same direction in the other species. This mouse-human conservation of gene expression gradients suggests that common structural features may shape hierarchical functional specialization in both mammalian brains.

Whereas our results demonstrate a surprising consistency in how transcriptional gradients vary with T1w:T2w, they also provide insights into key differences in the degree of specialization in the mouse cortex. Previous comparative studies of mouse and primate have focused on quantifying microstructural variation between areas with the most disparate function, e.g., between V1 and frontal cortex in mouse and between V1 and lateral prefrontal cortex in rhesus monkey.^20^ This research has painted a consistent picture of the relative uniformity of the mouse brain compared with the highly differentiated primate cortex. In our study, correlations between T1w:T2w and other spatial maps were consistently weaker in mouse relative to macaque and human: for hierarchical level (ρ_macaque_ = 0.76, ρ_mouse_ = 0.29), cytoarchitectural type (τ_macaque_ = 0.87, τ_mouse_ = 0.51), and the leading principal component of gene transcription (ρ_mouse_ = 0.53, ρ_human_ = 0.81).^11^ In characterizing the most salient mouse-human differences in brain structure, we also flagged candidate genes for further investigation that show the most difference between mouse and human, including NMDA receptor signaling genes *Grin2b*/*GRIN2B* (ρ_m_ = 0.19, ρ_h_ = –0.63), *Grin2d*/*GRIN2D* (ρ_m_ = –0.41, ρ_h_ = 0.13), and *Grin3b*/*GRIN3B* (ρ_m_ = –0.34, ρ_h_ = 0.26).

## Validating Gene Markers for Cell Types

**Figure 2.**
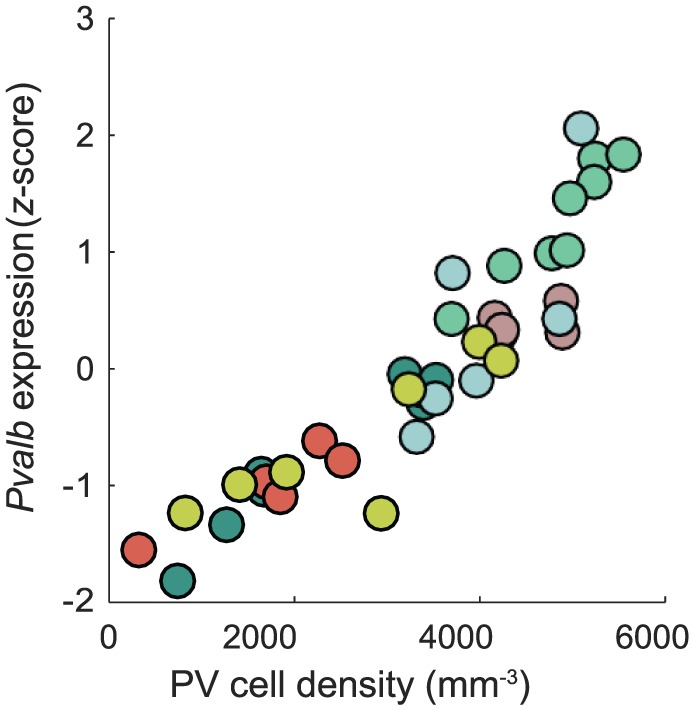
*Pvalb* expression is strongly correlated with an independent measure of parvalbumin containing cell density across the mouse cortex. We plot *Pvalb* expression (*z*-scored expression energy estimated from coronal section data from the Allen Mouse Brain Atlas^3^) as a function of direct quantitative measurement of parvalbumin containing cell density^4^ across cortical areas. The 2 measurements are highly correlated, Spearman ρ = 0.95, increasing from prefrontal and anterolateral areas through to somatomotor areas (brain areas are colored as labeled in Figure 1).

Bringing together measurements of gene expression^5^ with direct measures of interneuron cell densities^6^ allowed us to validate gene markers for interneuron cell types. We demonstrated that using gene expression data as a proxy for cell-type density can be highly accurate, as shown for the strongest correspondence we observed between the density of parvalbumin-containing interneurons^6^ and the independently measured expression of *Pvalb*^5^ across cortical areas in Figure 2. The correspondence for vasoactive intestinal polypeptide (VIP) cell density with *Vip* gene expression was also strong, ρ = 0.76 (*P* = 3 × 10^−7^), but was much weaker for somatostatin (SST) cell density with *Sst* expression, ρ = 0.24 (*P* = 0.1). The lack of agreement for *Sst* may be due to a breakdown in the relationship between *Sst* expression and SST cell density: *Sst* expression decreases in adults and is regulated by neural activity.^21^ Our results suggest that the inference of cell density from gene expression atlases can be accurate, but a direct correspondence can be confounded by the many factors regulating gene transcription, as well as additional factors complicating straightforward inference of protein levels from mRNA.^22^ Continuing the search for cell types and their marker genes,^23,24^ and validating them against independent cell density measurements,^6,7^ will equip us with tools to infer the spatial distributions of cell types, including their contribution to psychiatric phenotypes.

## Spatial Continuity of the Macroscopic Brain

At the macroscale, the brain is a continuous physical system^25^ that is commonly approximated as a set of discrete brain areas partitioned by hard boundaries. Our analysis brought together many open datasets of spatial maps reported at the level of the Allen Reference Atlas areas, but neuroscience datasets are now being measured and shared at cellular resolution.^26^ These data open the door to future research overcoming the limitations of discrete parcellations, and moving toward a continuous characterization of the fine-scale spatial patterning of the brain’s structural and functional properties. The next generation of brain atlases will likely contain a far more subtle representation of the brain’s spatial organization that takes into account multimodal information measured at each voxel,^27^ likely yielding a combination of continuous gradient-like variation^28^ as well as sudden changes consistent with interareal boundaries.

## The Need for Theoretical Frameworks

Linking neuroscience big data to scientific understanding requires statistical analysis to uncover and characterize interesting patterns and theories that explain diverse observations in a coherent conceptual framework.^1^ Seen through this lens, the multimodal statistical relationships we present in Fulcher et al.^4^ will be important for constraining new mechanistic models of brain dynamics that bridge spatial scales of brain organization by capturing whole-brain activity dynamics in terms of putative physiological mechanisms. Whole-brain models, whether formulated physically (e.g., neural fields) or phenomenologically (e.g., as coupled dynamical systems), have overwhelmingly made the simplifying assumption of spatially uniform parameters when simulating brain dynamics. New models are beginning to incorporate inter-regional heterogeneity, allowing model parameters to vary with an empirical measurement such as spine count^29^ or T1w:T2w.^30^ Models including this simple 1-dimensional heterogeneity yield better out-of-sample fits to experimental functional-connectivity measurements and the hierarchical organization of intrinsic timescales,^30^ providing evidence that hierarchical variation in local circuits plays a key role in shaping cortical dynamics. Physiologically based models, as opposed to phenomenological models, stand to gain the most from the rich new neuroscience datasets, as their formulation and parameters can be directly constrained and verified against data.^25^

The modern landscape of neuroscience has been shaped by advances in neuroinformatics, data-sharing standards, and greater contributions from statistical and computational modeling in generating guiding hypotheses for new experiments. Integrative studies like Fulcher et al.^4^ that assemble many diverse datasets across multiple species will play a key role in uncovering the principles of how the brain’s organization enables a diverse functional repertoire.
